# Homographic Patch Feature Transform: A Robustness Registration for Gastroscopic Surgery

**DOI:** 10.1371/journal.pone.0153202

**Published:** 2016-04-07

**Authors:** Weiling Hu, Xu Zhang, Bin Wang, Jiquan Liu, Huilong Duan, Ning Dai, Jianmin Si

**Affiliations:** 1 Department of Gastroenterology Sir Run Run Shaw Hospital, Zhejiang University, Hangzhou, China; 2 Institute of Gastroenterology, Zhejiang University, Hangzhou, China; 3 College of Biomedical Engineering & Instrument Science, Zhejiang University, Hangzhou, China; 4 Key Laboratory of Biomedical Engineering, Ministry of Education, Zhejiang University, Hangzhou, China; University Hospital Llandough, UNITED KINGDOM

## Abstract

Image registration is a key component of computer assistance in image guided surgery, and it is a challenging topic in endoscopic environments. In this study, we present a method for image registration named Homographic Patch Feature Transform (HPFT) to match gastroscopic images. HPFT can be used for tracking lesions and augmenting reality applications during gastroscopy. Furthermore, an overall evaluation scheme is proposed to validate the precision, robustness and uniformity of the registration results, which provides a standard for rejection of false matching pairs from corresponding results. Finally, HPFT is applied for processing in vivo gastroscopic data. The experimental results show that HPFT has stable performance in gastroscopic applications.

## 1. Introduction

Registration of endoscopic images plays an increasingly important role in endoscopic surgeries [1]. For example, the registration technique can be applied to real 3D depth recovery [2], lesion tracking [3, 4], endoscopic image mosaicking [5], and other augmented reality view generation [6]. Registration techniques can also be utilized to guide interventions in minimally invasive surgery (MIS), such as reducing post-surgical trauma and reducing recovery time for patients [7]. Moreover, image registration is widely used in biopsy monitoring [8], follow-up examination [9] and therapy planning [10].

Many researchers have focused on medical image registration in the past few decades. Mikolajczyk [11] evaluated the performance of different image registration detectors in the computer vision field. Oliveira [12] made comments on the main contributions, advantages and drawbacks of classical and novel medical image registration methods. Despite the development of a wide range of registration methods, most of the current registration methods are applicable only in static scenes (e.g., sinus surgery and skull surgery) or scenes with periodic deformations [13]. In static scenes, the transformation between endoscopic images is caused by the motion of the camera, which may be considered global rigid motion. The motion is estimated by reliable corresponding features [14]. For periodic deformation scenes, several methods have been proposed to estimate the periodic parameters of the motion, which were used as motion compensation to extend the registration method from a static scene to a periodic deformation scene [15]. Although deformable registration methods have been developed in recent years [16], their direct application to free-form tissue deformation remains an unsolved problem due to complicated motion and changing visual appearances [17, 18].

Additionally, there are also some difficulties and under-determined problems in gastroscopic image registration. First, gastric inflation and endoscopic interaction can lead to serious deformation of the stomach, which is usually problematic for the subsequent registration procedure in which the features of interest will change dramatically or disappear from the endoscope view [19]. Second, the effects of specular reflection, the shadow appearances and the paucity of reliable salient features lead to difficulties in detecting accurate features for image registration. Several studies have focused on endoscopic image registration and tracking by optical flow [20], which may result in an incorrect registration because of non-normalized lighting conditions. Some other studies attempted to utilize an illumination invariance feature detector in endoscopic images [21], but the detected sparse features were not suitable for real clinical practice. Third, endoscopists may operate the endoscope with a large twisting angle, which leads to content discontinuity in successive image sequences and makes registration even more difficult. Some researchers attempted to solve this problem by marking anatomical landmarks (e.g., skin markers, screw markers, dental adapters, etc.) or by introducing extrinsic features, rigidly positioned with respect to the patient [22]. However, these are not automatic methods and are not widely used.

Although gastroscopic image registration is challenging, it is one of the key links for computer aided diagnosis (CAD). For example, with accurate and robust matching results, lesion tracking can be developed during surgery [23], which is very helpful for intraoperative localization and navigation. Furthermore, the narrow field of view (FOV) of a gastroscope always limits gastroscopist operations. With accurate and density matched points, researchers can reconstruct gastric internal surfaces in a 3D view and panoramic view, which can provide a sufficient FOV for gastroscopists [2, 24].

The goal of this study is to develop a new registration method (named Homographic Patch Feature Transform (HPFT)) that can detect features in gastroscopic image sequences with robustness, precision and uniformity. Considering the smooth surface of the stomach, we assume that the points in a local patch of the gastric internal surface share a common plane in the real world. With this homographic hypothesis, these patches can be detected using HPFT. Moreover, if the local patches are not under the condition of homographic theory, a patch-split scheme can be performed to detect further homographic relationships in an iterative way. Lastly, an overall evaluation scheme is proposed to validate the precision, uniformity and robustness of HPFT by comparison with other registration methods.

In the authors’ opinion, HPFT offers three main contributions. First, the problem of corresponding gastroscopic images being presented densely can be solved with HPFT, which is very important for clinical practice (e.g., MIS, non-invasion biopsy, and virtual gastroscopy). Second, this method can be directly applied to currently implemented gastroscopy devices without any extra instruments (e.g., position sensors and feature markers). Third, HPFT can be applied to other abdominal or thoracic soft tissue organs (e.g., heart, lung, and liver), which also have smooth surfaces.

## 2. Methods

### 2.1 Method Overview

**Fig 1:**
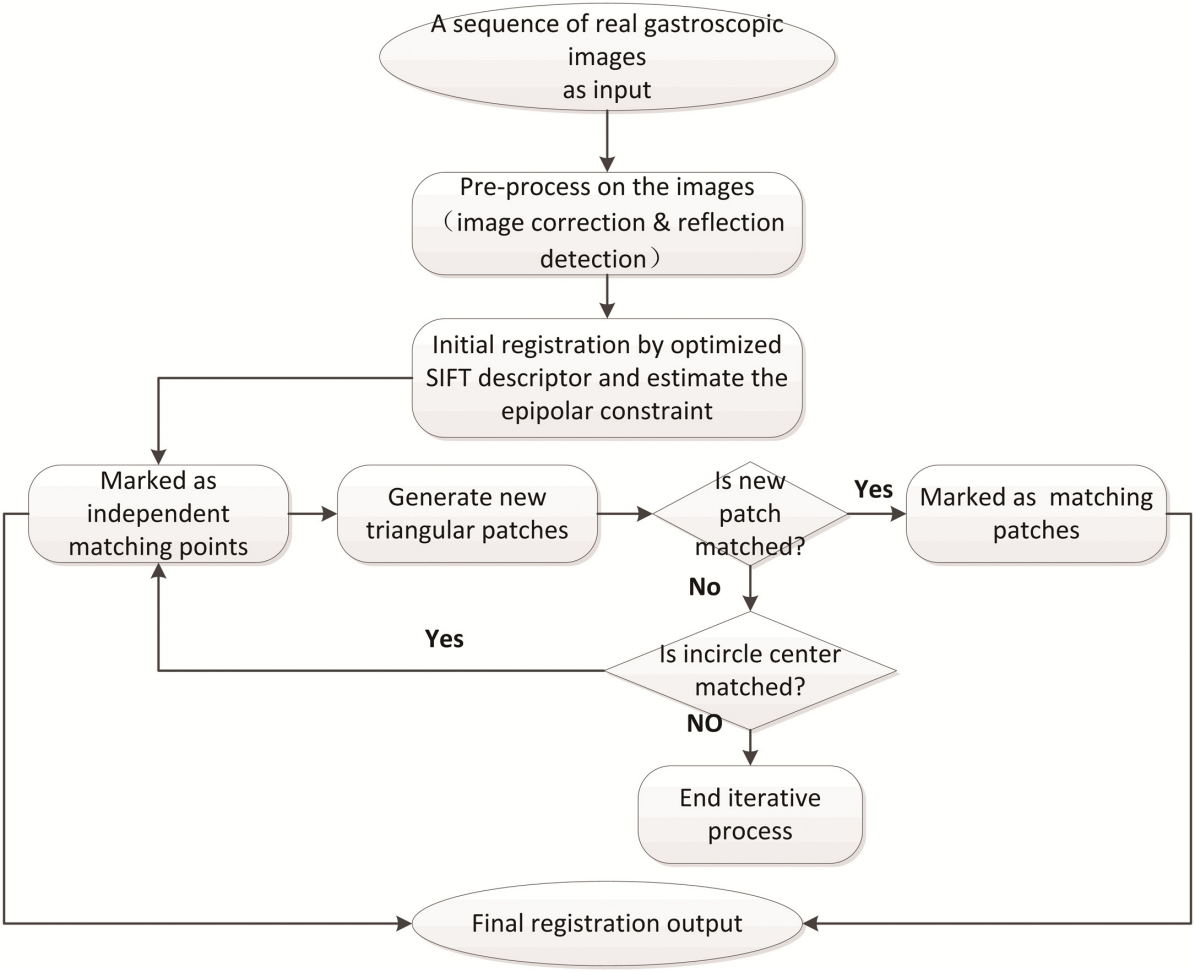
HPFT workflow.

The processing flow of HPFT is shown in Fig 1. The inputs are sequences of real gastroscopic images. The large distortion of the radial and tangential lens in the endoscope needs to be rectified with a camera calibration method before acquisition of the gastroscopy sequences, and the reflection region should be detected before registration.

To obtain the initial feature set and generate local patches, the gastroscopic images are processed by a suitable point feature-based method. In this study, some widely used feature detectors are evaluated, and the Scale Invariance Feature Transform (SIFT) local descriptor is adopted due to its excellent performance in illumination and scale changes [25]. Subsequently, the epipolar constraint is calculated, and then the initial matching pairs are clustered into small groups by the Delaunay Triangulation method. Each triangle represents a local patch on the gastric internal surface.

We hypothesized that the gastric internal surface is composed of many small homographic patches, and an iterative matching detection algorithm is proposed to detect triangular patches and verify whether they meet the homographic theory or not. If yes, these patches are marked as matching patches. Otherwise, they are split into smaller patches by their internal circle centers and are then verified with the homographic hypothesis in the next loop.

### 2.2 Pre-processing and Initial Feature Detection of Gastroscopic Images

To obtain a sufficient field of view during examination, gastroscopes are always equipped with fisheye cameras. Unfortunately, the generated gastroscopic images differ from their actual appearance due to serious distortion, as described in reference [26]. In this study, Zhang’s camera calibration method [27] is employed and improved [2] to correct the distorted images.

The method starts with establishing initial correspondences between gastroscopic image sequences. Because the endoscope can move flexibly in the stomach and acquire images at any viewpoint, the adopted descriptor should be robust with respect to rotation and scale. Some commonly used point feature methods (SIFT, FAST, SURF, and STAR) are estimated, and an estimation framework named Forward-Backward error (FB error) [28] is applied to select the most suitable detection method. During the estimation, the selected registration methods are applied to the first frame of the native gastroscopic images, and the detected features are matched from the first frame to the last frame. Afterwards, the features are detected and matched from the last frame to the first frame in reverse. Finally, a feature’s location will be the same as its initial location in the first frame if it was detected and matched accurately. Otherwise, the FB error is calculated as the deviation between the initial location and the tracked location.

We processed the estimation and showed the results in [23], proving that SIFT has higher accuracy than other methods. Thus, we adopt SIFT as the initial registration method to detect initial point features for further detection. To accelerate performance, we simplify the original SIFT vector to a 32 element vector and implement it in the GPU architecture.

### 2.3 Homographic Registration

The initial matching pairs are clustered into small groups using Delaunay triangulation, and each matching triangle contains three matching pairs. Luong and Faugeras [29] suggested that if two sets of the image points, m and m’, are the projections of a 3D plane in space, the matching relationship can be represented as a homographic transformation and can be conducted as:

m′=ρHm (1)

Where ρ represents an arbitrary non-zero scalar, and H is a 3*3 matrix.

The registration approach assumes that the area of the triangular patch is small enough to be consistent with the homographic theory, in which the H matrix has only six unknowns [30]. Under the homographic assumption, H can be estimated by three vertices of the patches. Once, H is determined, every point in the patch of an image should be wrapped and aligned in the corresponding patch of the other image. Normalized cross correlation (NCC) contributes to validation of the homographic assumptions.

Considering (v1, v2, v3) as the coordinates of the vertex of the patch, all interior points of the triangle can be represented in barycentric coordinates. Once a point in the reference triangle is given by (s, t), an interior point’s coordinate in the patch can be presented as:

$$p(s,t)=sv_{1}+tv_{2}+(1-s-t)v_{3}$$ (2)

Where 0<s <1, 0< t<1, s + t<1.

The intensity value of the point (s, t) is described as I(s, t). The validation of the homographic hypothesis for each matching patch is defined as the NCC of the intensity:

$$Dif_{H}=\frac{\int_{s=0}^{1}\int_{t=0}^{1}I(s,t)I(\rho Η(s,t))}{\int_{s=0}^{1}I(s,t)\int_{t=0}^{1}I(\rho Η(s,t))}$$ (3)

The value of *Dif*_*H*_ represents a similarity of two matching patches. The closer the value is to 1, the higher the similarity of the two matching patches and the more reasonable the homographic assumption of the matched patches. In contrast, if the *Dif*_*H*_ is far away from 1, the interior pixels of the triangular patches cannot be matched. The most common case is that the matching triangle is not small enough, so the corresponding gastric internal surface cannot be considered a plane in the real 3D space.

### 2.4 Iterative Registration

For clarity, we name the matching image pair the reference image and the target image. The iterative process can be described as follows:

#### Step 1

The features and matching pairs are detected between the reference image and the target image, and the matching results are recorded in the matching points set.

#### Step 2

The matching points are clustered into triangular patches, and the patches are matched by the method described in section 2.3. The matching patches are recorded in the matching patches set.

#### Step 3

**Fig 2:**
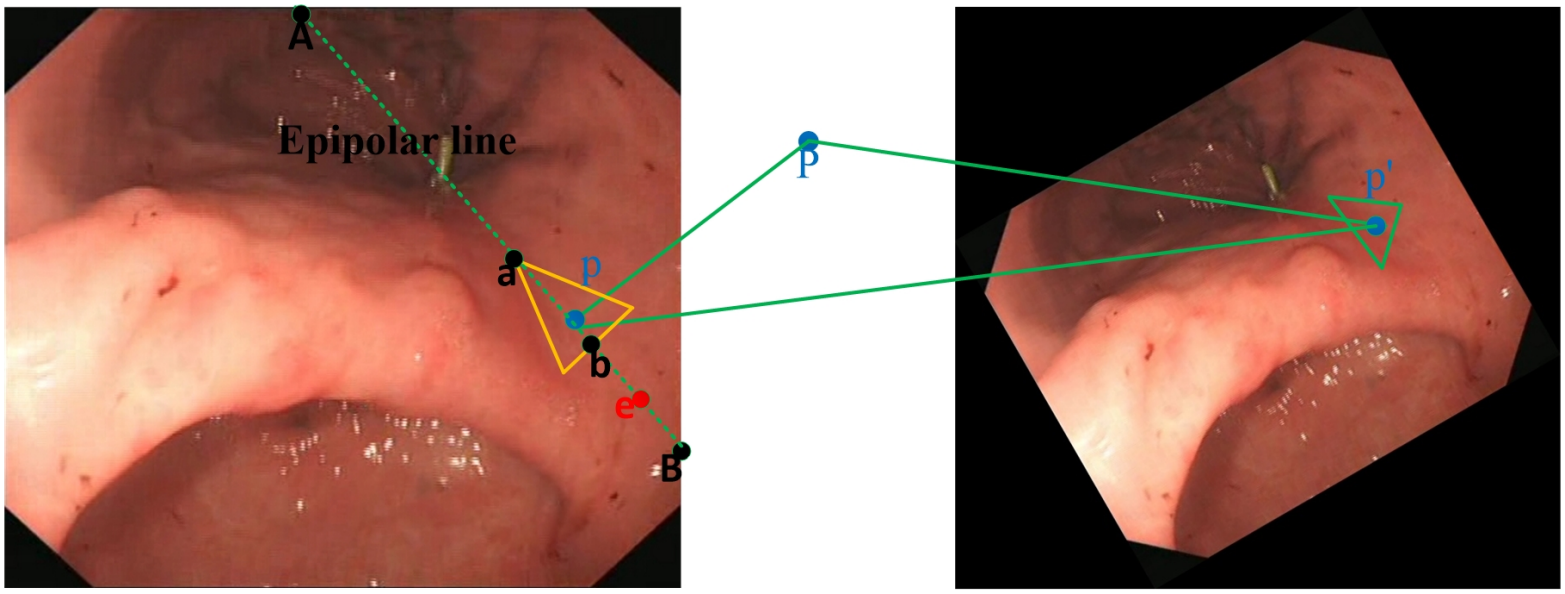
The search line (from a to b in the left image) determined by epipolar constraint and homographic constraint.

Patches that cannot be matched in Step 2 are selected, and their internal circle center points in the reference image are marked. Subsequently, the center points are matched with the points on the line determined by the epipolar line and the corresponding triangular region in the target image (Fig 2). The estimation of the matching pairs is described as the Euclidean distance of the points’ SIFT feature:

$$V_{i}=\sqrt{\sum_{i=1}^{32}{\left(e_{i}-e_{{}_{i}}^{\prime }\right)}^{2}}$$ (4)

Where e_i_ and e_i_’ represent two normalized vector elements of a matching pair’s SIFT descriptor in the reference image and target image, respectively.

#### Step 4

During the process of matching a pixel in the reference image with the points on the line segment in the target image, the alterative matching results are considered a vector: [V_1_, V_2,_ V_3_ … V_i_] (i represents the total point number on the line segment). The most reliable matching pair should meet two conditions:

1. If V_min_/V_second-min_>0.8, there is no real matching pair due to the uncertainty of the matching process.
2. Suppose Ɛ is a global parameter for evaluating V_min_, and only when V_min_<Ɛ will the corresponding possible matching pair be considered a real matching pair.

$${\left(V_{1},V_{2},V_{3},\ldots V_{i}\right)}_{\text{min}}<\varepsilon$$ (5)

The Ɛ can avoid misjudging a corresponding pair when there is no matching pair.

#### Step 5

The internal circle center points of un-matched patches in the target image are selected and matched to the reference image reversely, which is a symmetrical operation of Step 3 and Step 4.

#### Step 6

The matching points are recorded in the matching pairs set. Step 2 is repeated until no new matching points and no new matching patches are detected.

## 3. Matching Evaluation

### 3.1 Evaluation Overview

The registration results include the matching points and matching patches. It is difficult to evaluate the matching results due to the absence of a gold criterion in an endoscopic environment. Some studies undertake evaluations by human assessments; for example, a feature matching method used in MIS was also proposed in [31], and the matching results were evaluated by clinical experts. Thus, the clinical experts’ interventions ensured that the final assessments were in accordance with real clinical requirements, which is of great significance to endoscopists and real clinic practice. However, human assessments are not suitable for large amounts of test data. In this study, HPFT is aimed at applications in future image guided technologies (gastroscopic image mosaicking and gastric depth recovery) in which precision, uniformity and robustness are very important factors. Consequently, an overall matching evaluation scheme to assess the precision, uniformity and robustness of the registration findings is presented.

### 3.2 Precision Estimation

Precision estimation primarily evaluates inaccurate detections in the registration results. The main problem for precision estimation is related to the lack of ground truth data. Here, we use a Kullback-Leibler divergence (D_KL_) method to evaluate the matching FB trajectory error.

In information theory, Kullback-Leibler divergence is a measurement of the difference between two probability distributions. For example, the D_KL_ of Q from P, denoted by D_KL_(P||Q), is a measurement of information loss when Q is an approximation to P, and D_KL_ is a non-symmetric operation. To evaluate the precision of HPFT, the distributions of the matching pairs in forward and backward trajectories are considered discrete random variables and defined as P_Forward_ and P_Backward,_ respectively. The distributions are compared by measuring the Kullback-Leibler divergence:

$$D_{KL1}(P_{Backward}(x)||P_{Forward}(x))=\sum_{x\in X}P_{Backward}(x)ln[\frac{P_{Backward}(x)}{P_{Forward}(x)}]$$ (6)

$$D_{KL2}(P_{Forward}(x)||P_{Backward}(x))=\sum_{x\in X}P_{Forward}(x)ln[\frac{P_{Forward}(x)}{P_{Backward}(x)}]$$ (7)

The variable x in eqs (6) and (7) denotes the coordinates of the matching pairs. According to the definition of the forward-backward method, if the matching precision is high, the matching trajectories in the forward process and backward process should be similar, and the D_KL_s are small and of the same order.

### 3.3 Robustness Estimation

In statistical analysis, the covariance matrix can represent the correlation of variables. Zhang, Hartley and Zisserman et al. [32] found that the covariance matrix can characterize the uncertainty of the fundamental matrix. As a derivation, Baptiste Allain et al. [33] employed this method to determine the accuracy of tracking the biopsy site under different noise environments. In the subsequent analysis, the covariance matrix of the matching site is used to estimate the robustness of the registration method.

A pixel in the reference image is denoted as p, and the registration site in the target image is denoted as p′. In different registration scenes (e.g., different noise and different brightness), p′ may be located in different sites, and the covariance matrix of p′ is defined as:

$$Cov_{p\prime }=E[(p\prime -E[p\prime ])\cdot (p\prime -E{\left[p\prime \right]}^{T})]$$ (8)

If p' can be represented by (x’, y’), the following equation can be derived as:

$$Cov(x\prime ,y\prime )=\left(\begin{array}{cc} {\text{var}}_{x\prime x\prime } & {\text{cov}}_{x\prime y\prime }\\ {\text{cov}}_{x\prime y\prime } & {\text{var}}_{y\prime y\prime } \end{array}\right)$$ (9)

According to the well-known large number law in the statistical field, if we assume a large number of samples of p′ exist, E[p’] can be approximated by the sample mean:

$$E_{m}[p\prime^{\left(k\right)}]=\frac{1}{M\cdot \sum_{k=1}^{M}p\prime^{\left(k\right)}}$$ (10)

where M is the number of the samples set, p′^(k)^ is the k^th^ sample in the set.

The covariance matrix can be approximated by (11):

$$Cov_{p\prime }=[\frac{1}{\left(M-1\right)}]\cdot \sum_{k=1}^{M}\left[(p\prime^{\left(k\right)}-E_{m}[p\prime^{\left(k\right)}])\cdot {\left(p\prime^{\left(k\right)}-E_{m}[p\prime^{\left(k\right)}]\right)}^{T}\right]$$ (11)

During the gastroscopy procedure, if the registration algorithm is run M times in each acquired image, the covariance of p′ can be computed statistically, and it can be considered a robust registration method in endoscopic surveillance. However, it requires the endoscope to remain still during the running process. Obviously, this is not practical during an endoscopic procedure. To estimate the uncertainty statistically, a series of standard Gaussian noises are added with different scalars to the original gastroscopy images for M times as a simulation.

## 4. Experiments and Results

**Fig 3:**
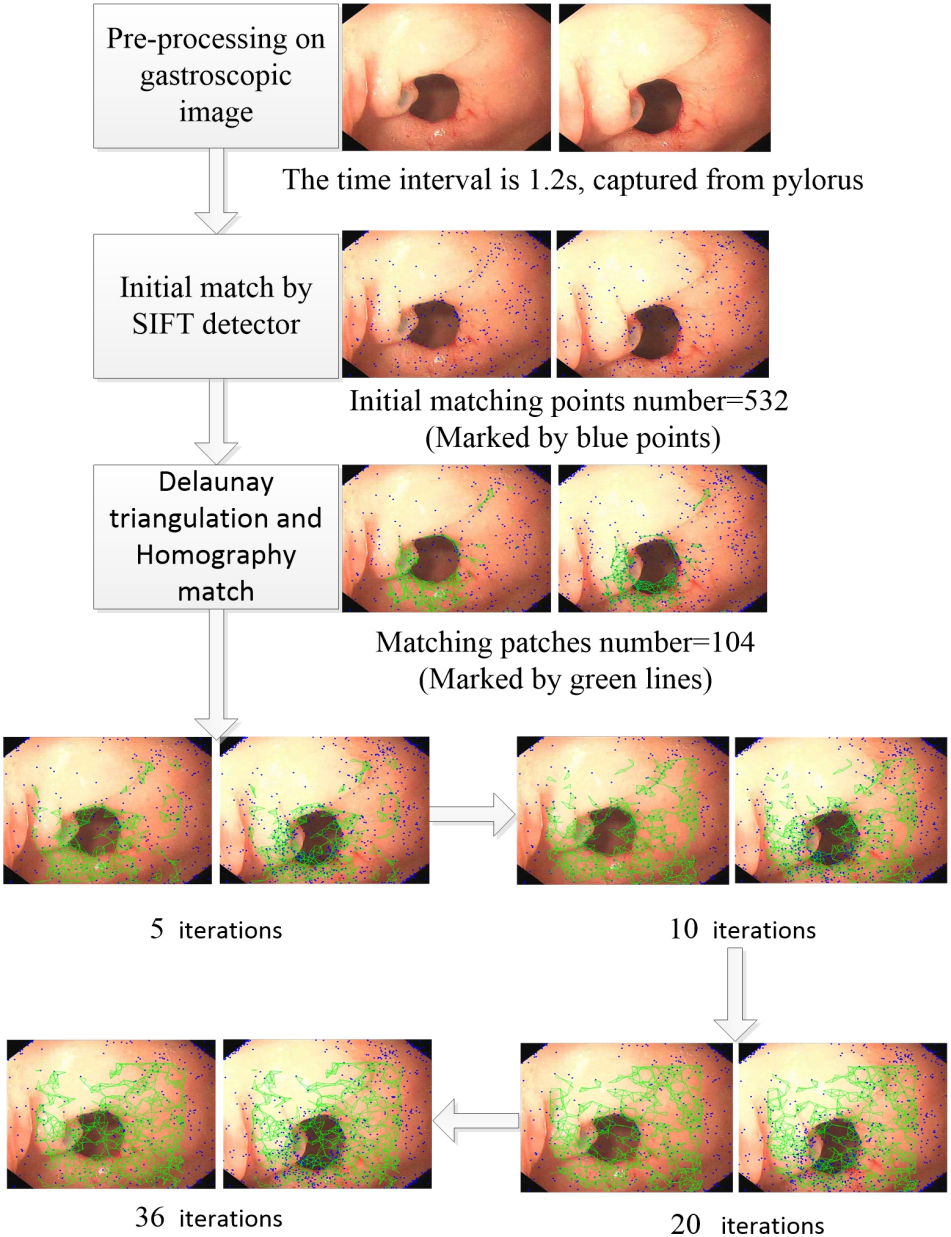
Workflow of the registration on the pylorus. HPFT finished after 36 iterations. The blue points indicate matching points, and the green triangles indicate matching patches.

To evaluate the performance of our method in real gastroscopy procedures, the method was applied to the real gastroscopy image data from Sir Run Run Shaw Hospital in Zhejiang province in China, and the matching results were compared with other registration methods. All 60 patients provided written informed consent for evaluation and follow-up using medical records. Our research was approved by the Ethics Committee of Sir Run Run Shaw Hospital, School of Medicine, Zhejiang University. The gastroscopy video was acquired at 25 fps. To ensure confidentiality, the examination information (e.g., examination date and patient’s name) was removed from the original gastroscopy images. The processed image sizes were 560*480. Fig 3 shows the workflow for the pyloric sites by the proposed method.

To evaluate the uniformity of the registration results, we selected seven gastroscopy sequences randomly, and each of them was acquired from an anatomical site (pylorus, cardia, angularis, antral anterior wall, antral posterior wall, lesser curvature of the gastric body, and greater curvature of the gastric body). First, HPFT and the other registration methods were applied to detect features from the first frame of the seven gastric sequences. Then, the features were matched forward to the last frame and then matched from the last frame to the first frame. The features’ tracking trajectories were estimated.

### 4.1 Effectiveness of the Evaluation Scheme

We evaluated the registration result by the proposed evaluation scheme. As a consequence, the effectiveness of the evaluation scheme was demonstrated. The common method for this entails applying the evaluation scheme to existing datasets, which have inherent validity for the matching process. Thus, by comparison with the existing datasets, we could draw a conclusion on the proposed scheme.

There are several public matching evaluation datasets; for example, in [34], an available image database is provided, and the samples in this dataset contain rotation and deformation that can be used for image classification, recognition and other image processing tasks; however, this image dataset does not contain endoscopic image cases. In [35], an endoscopic image dataset is provided, however, the images were used in laparoscopic cases, and they are not suitable for a gastroscopy environment.

As a result, we estimated an effective evaluation framework, and we took advantage of the gastroenterologists’ experiments to make the estimation. First, HPFT was applied to the test data, and we evaluated the matching results with the proposed evaluation scheme and employed three experienced gastroenterologists separately. The gastroenterologists provided credible matching evaluations, from which we computed the recall percentage and accuracy percentage of our automatic evaluation scheme (Table 1).

**Table 1:** The recall and precision percentages of the pvaluation.

	pylorus	cardia	angularis	antral posterior wall	gastric body lesser curvature	gastric body greater curvature
**recall**	0.85	0.88	0.81	0.87	0.91	0.90
**precision**	0.92	0.95	0.87	0.92	0.94	0.88

As can be seen in Table 1, the recall percentage and precision percentage were both larger than 0.8, which indicated the proposed matching evaluation rationality. Compared with the gastroenterologists’ evaluations, the proposed evaluation scheme can run automatically, and in Section 4.2, we evaluate HPFT matching results using the proposed evaluation scheme.

### 4.2 Automatic Matching Evaluation

During the experiment, the number of the initial detected features for the test methods was limited to 200. If the number of the initial features was larger than 200, we randomly selected 200 features as the initial detected set. Moreover, if the initial detected features were located freely in the reference images, the features may be missing in the target image due to changes in the endoscope’s view during gastroscopy. To solve this problem, before the experiment, we set a region of interest (ROI) in the first frame, ensuring that the ROI appeared in all of the following frames, and also ensured that the initial features in the ROI were detected with all registration methods. The testing image sequences were encoded with an MPEG2 standard; the duration was 60.0 s, and the frame number was 1,500. Because the content of adjacent frames was very similar, we did not need to match all of the frames of the testing image sequences. In this experiment, only the I and P frames were considered testing frames, and the actual testing frame number was 500.

Before evaluating the D_KL_ of the feature tracking trajectories, we estimated the FB error of the feature point trajectories. We considered the distance of FB errors lower than 4 pixels to be reliable pairs. The matching result’s precision percentage was defined as in (12), and Table 2 shows the FB error curves for the testing sequences.

$$\text{Pr}ecisionPercentValidation(PPV)=\frac{MatchingNumber(FBerror\leq 4)}{InitialFeatureNumbers(200)}$$ (12)

**Table 2:** The precision percent validation (PPV).

	HPFT	ORIGINAL SIFT	FAST	SURF	STAR
**pylorus**	0.85	0.63	0.33	0.57	0.51
**cardia**	0.83	0.65	0.31	0.52	0.47
**angularis**	0.82	0.78	0.42	0.67	0.53
**antral anterior wall**	0.62	0.47	0.28	0.53	0.34
**antral posterior wall**	0.77	0.43	0.37	0.47	0.34
**gastric body lesser curvature**	0.76	0.59	0.11	0.32	0.27
**gastric body greater curvature**	0.69	0.57	0.25	0.41	0.43

It can be seen from Table 2 that HPFT was significantly better than the other registration methods. The second best method was the original SIFT, which was not simplified by the method proposed in this study. Compared with the original SIFT’s PPV, HPFT had an improvement of more than 70% in the antral and gastric body, which illustrated that the surface of the anatomical site was flat and that HPFT had good performance in detecting homographic matching. For an angularis site, the fold and bending made homographic matching difficult, and the improvement was not significant, which was consistent with results shown in Table 2.

We found that the least time was required by FAST on average and that it detected as many matching pairs as HSIFT and SIFT for some anatomical sites. Unfortunately, almost 60% of the matching pairs were false pairs. In addition, HPFT ran much faster than SIFT (for a 560*480 image, HPFT took 0.3 s, and SIFT took 1.1 s) and the matching results were good. This indicated that the simplified SIFT had a reasonably faster performance than the original method, without loss of accuracy.

Although FB error was an easily implemented estimation method, an additional precision analysis should be utilized to evaluate the FB error estimation’s confidence. As explained in section 3.1, we applied D_KL_ to HPFT’s feature estimation results, which were evaluated by FB error. Every feature’s tracking trajectory was considered a random distribution, and the forward distribution and backward distributions were identical if D_KL1_<0.1, D_KL2_<0.1 and D_KL1_ and D_KL2_ were of the same order.

In Table 3, the D_KL_ column indicates estimations by D_KL_ on the FB error estimation results; the ‘FB error’ column indicates estimations by FB error, and it corresponded to the ‘HPFT’ column in Table 2. The ‘D_KL_ / FB error’ column indicates the similarity of the D_KL_ estimation and FB error estimation. If the two estimation results were almost identical, the value of ‘D_KL_ / FB error’ should be close to 1, and the precision estimation of HPFT was considered reliable. Otherwise, the two estimation results were totally different and the precision estimation was suspect. As seen from Table 3, all ‘D_KL_ / FB error’ values of the testing image sequences’, except for ‘Angularis’, were higher than 0.9, and we considered the precision of HSIFT for these anatomical sites reliable. However, the ‘D_KL_ / FB error’ of ‘Angularis’ was 0.79, indicating that the precision of HSIFT for ‘Angularis’ was not very high. In the authors’ opinion, this was due to the narrow nature of the angularis. When the endoscope acquired images from the angularis, the content change was larger than expected between frames. In this case, HPFT had a higher probability of false matching. However, compared with other methods, HPFT was also more reliable in identifying precision matching pairs for the angularis.

**Table 3:** D_KL_ estimation of HPFT for different anatomical sites.

	D_KL_	FB ERROR	D_KL_ / FB ERROR
**pylorus**	165	170	0.97
**cardia**	154	166	0.93
**angularis**	131	164	0.79
**antral anterior wall**	118	124	0.95
**antral posterior wall**	142	154	0.93
**gastric body lesser curvature**	139	152	0.91
**gastric body greater curvature**	125	138	0.91

To evaluate the robustness of HPFT, we added a series of Gaussian noise to the gastroscopy image sequences and evaluated the robustness of the precision estimation results. The most robust registration method should generate the most identical registration results under different Gaussian noise environments, and the consistency can be evaluated by the covariance matrix, as expressed in eq (11). If the covariance matrix (Cov_p’_) was close to one, the registration results were not violated by the noise, and we considered Cov_p’_>0.8 a robust registration result. For different methods, the robustness percent validation (RPV) was defined as:

$$Robustness\;Percent\;Validation(RPV)=\frac{MatchingNumber(Cov\geq 0.8)}{PPV}$$ (13)

**Fig 4:**
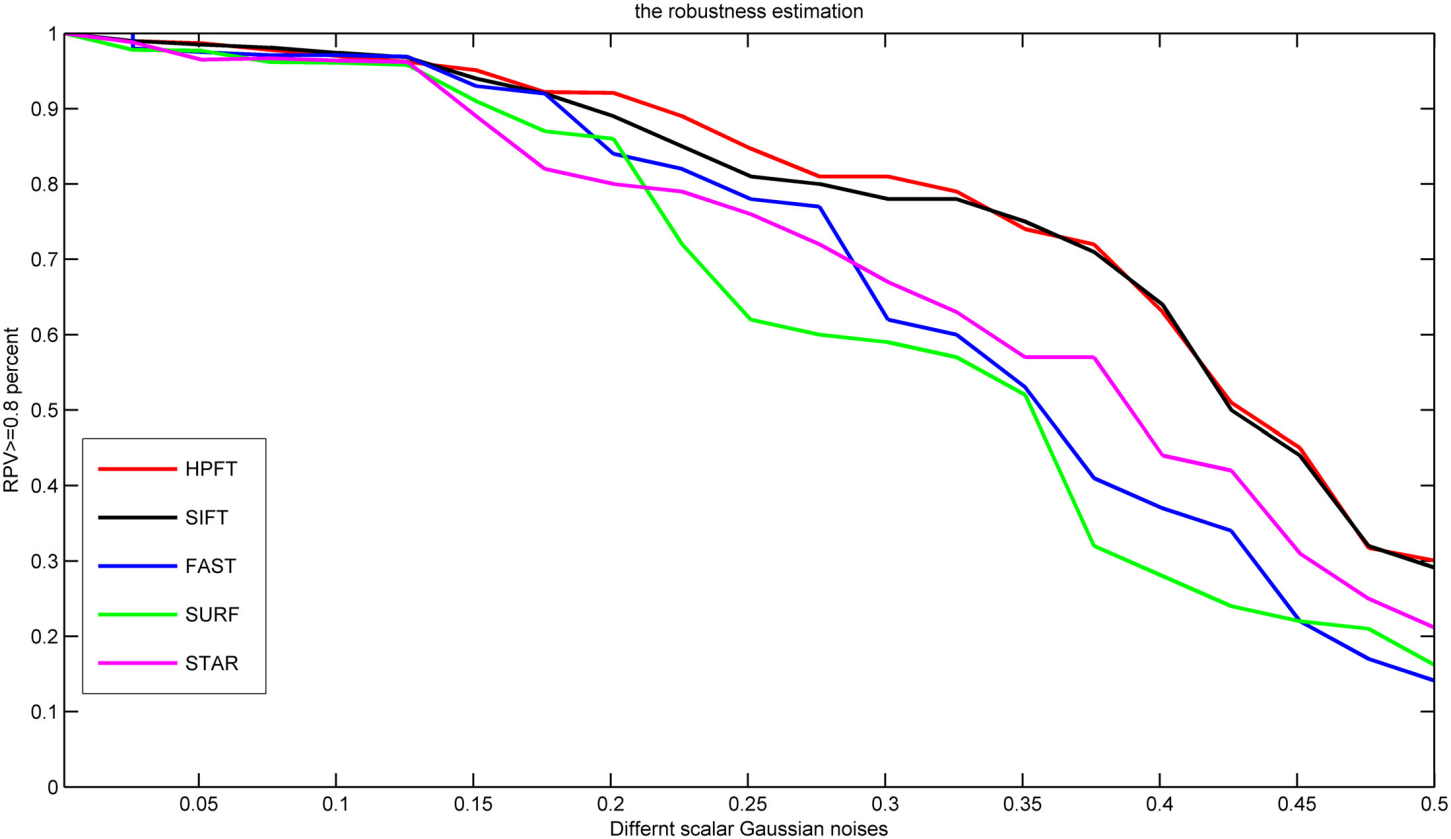
Robustness estimation for different registration methods. RPV>0.7 was considered robust (dashed line).

RPVs for different registration methods are shown in Fig 4. It can be seen from Fig 4, that HPFT and SIFT had similar robustness estimation results. The other registration results had poorer robustness than SIFT and HPFT.

**Fig 5:**
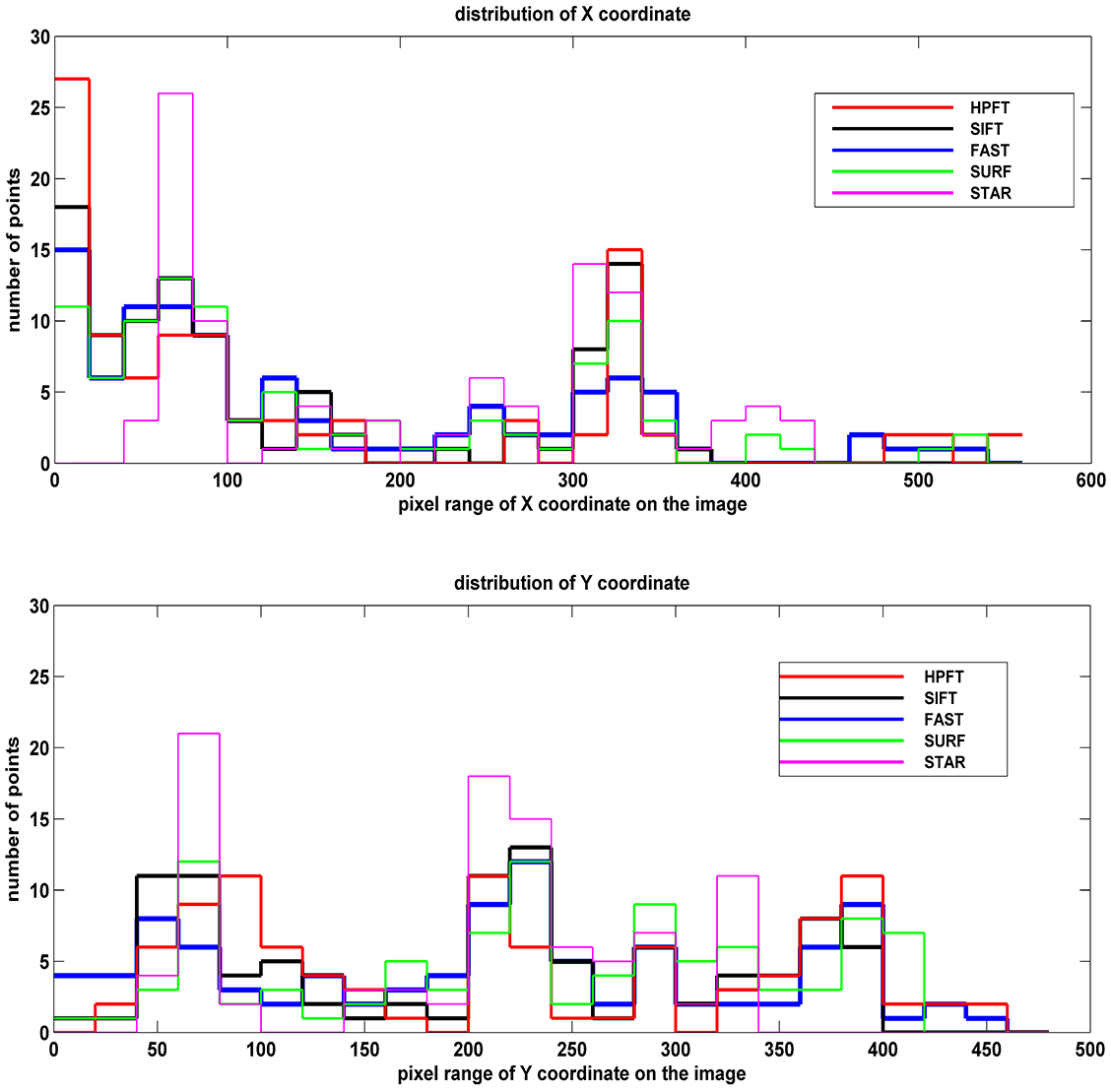
Initial feature distributions of X and Y coordinates. Because the initial test feature number for all registration methods was 200, obviously, the lower squared difference value corresponded to the more uniform distribution.

The uniformity of the matching results was assessed by the squared difference of the matching pair coordinates. The statistics of 200 initial feature distributions by different registration methods are shown in Fig 5.

The squared differences are shown in Table 4.

**Table 4:** The squared difference of registration results.

	HPFT	SIFT	FAST	SURF	STAR
**pylorus**	15.02	19.14	34.28	14.19	32.61
**cardia**	8.79	10.17	13.3	12.11	36.53

As seen in Table 4, HPFT and SURF had reasonable uniformity, and FAST and STAR’s features were relatively concentrated.

## 5. Conclusion

In this study, an iterative method for registration during gastroscopic processes was presented. A local feature descriptor was used to detect the initial matching pairs. Epipolar geometry and homographic transformation were considered for further matching based on the initial matching pairs. The final registration results were composed of the independent matching point pairs and the matching patches. An overall estimation method was proposed to determine the precision of the registration. Experimental results using real gastroscopic images showed that the method has promising performance ability.

The gastric internal surface is always covered with mucus, so matching errors will easily occur from the specular reflection. The integration of other visual cues, such as shading, in response to soft-tissue deformation can improve the registration results. Another limitation of this method is that the transformation between patches is always considered to be a homographic transformation. In the future, non-rigid transformations (e.g., radial basis function kernel) should be considered to exploit more potential matching patches.
